# Implementation and Impacts of California Senate Bill 1152 on Homeless Discharge Protocols

**DOI:** 10.5811/westjem.60853

**Published:** 2023-11-08

**Authors:** Haruna Aridomi, Yuri Cartier, Breena Taira, Hyung Henry Kim, Kabir Yadav, Laura Gottlieb

**Affiliations:** * University of California San Francisco, School of Medicine, San Francisco, California; † Social Interventions Research Evaluation Network, San Francisco, California; ‡ Olive View-UCLA Medical Center, Department of Emergency Medicine, Sylmar, California; § Harbor-UCLA Medical Center, Department of Emergency Medicine, Torrance, California; ∥ The Lundquist Institute for Biomedical Research, West Carson, California; ¶ University of California San Francisco, Department of Family and Community Medicine, San Francisco, California

## Abstract

**Introduction:**

In recent decades, there has been a growing focus on addressing social needs in healthcare settings. California has been at the forefront of making state-level investments to improve care for patients with complex social and medical needs, including patients experiencing homelessness (PEH). Examples include Medicaid 1115 waivers such as the Whole Person Care pilot program and California Advancing and Innovating Medi-Cal (CalAIM). To date, California is also the only state to have passed a legislative mandate to address concerns related to the hospital discharge of PEH who lack sufficient resources to support self-care. To this end, California enacted Senate Bill 1152 (SB 1152), a unique legislative mandate that requires hospitals to standardize comprehensive discharge processes for PEH by providing (and documenting the provision of) social and preventive services. Understanding the implementation and impact of this law will help inform California and other states considering legislative investments in healthcare activities to improve care for PEH.

**Methods:**

To understand health system stakeholders’ perceived impact of SB 1152 on hospital discharge processes and key barriers and facilitators to SB 1152’s implementation, we conducted 32 semi-structured interviews with key informants across 16 general acute care hospitals in Humboldt and Los Angeles counties. Study data were coded and analyzed using thematic analysis informed by the Consolidated Framework for Implementation Research.

**Results:**

Participants perceived several positive impacts of SB 1152, including streamlined services, increased accountability, and more staff awareness about homelessness. In parallel, participants also underscored concerns about the law’s limited scope and highlighted multiple implementation challenges, including lack of clarity about accountability measures, scarcity of implementation supports, and gaps in community resources.

**Conclusion:**

Our findings suggest that SB 1152 was an important step toward the goal of more universal safe discharge of PEH. However, there are also several addressable concerns. Recommendations to improve future legislation include adding targeted funding for social care staff and improving implementation training. Participants’ broader concerns about the parallel need to increase community resources are more challenging to address in the immediate term, but such changes will also be necessary to improve the overall health outcomes of PEH.

Population Health Capsule What do we already know about this issue?

*In 2019 California enacted Senate Bill 1152 (SB1152), a novel hospital mandate to standardize discharge protocols for patients experiencing homelessness.*

What was the research question?

*We explored the law’s implementation facilitators and barriers, and impact on hospital discharge protocols.*

What was the major finding of the study?

*SB1152 helped systematize discharge protocols, but had implementation barriers.*

How does this improve population health?

*Findings can inform future legislative efforts to improve health care services for patients experiencing homelessness.*

## INTRODUCTION

In the context of compelling evidence that social and economic circumstances influence health and healthcare utilization, healthcare systems are increasingly exploring ways to address adverse social determinants of health. Much of the attention in this evolving area has centered on improving care for patients experiencing homelessness (PEH), since homelessness is strongly associated with barriers to healthcare access, worse physical and mental health outcomes, increased mortality, and higher healthcare utilization costs.^1^ ^,^ ^2^ For example, various healthcare screening tools have emerged to assess homelessness and other social needs in clinical settings.^3^ In some states, screening and documentation of homelessness have been incentivized with reimbursement models that risk-adjust payments based on social adversity.^4^ Beyond screening, other healthcare investments have focused on care coordination and discharge planning for PEH. Some initiatives such as Chicago’s Better Health through Housing and the national Healthcare for the Homeless program have shown improved patient health outcomes and decreased hospital costs.^5^ ^,^ ^6^

In California, several state initiatives have been implemented in an attempt to improve care for patients with complex social and medical needs, including PEH. These include successive Medicaid 1115 waivers such as the Whole Person Care (WPC) pilot programs and California Advancing and Innovating Medi-Cal (CalAIM).^7^ ^,^ ^8^ Studies of the WPC pilots, which in many participating counties were targeted to PEH, showed reduced healthcare expenditures, decreased readmission rates, improved availability of services, and improved mental health of participants.^9^ Despite these state-level investments, concerns have persisted about PEH being discharged from acute care settings without sufficient resources to maintain wellbeing,^10^ with recent high-profile media coverage drawing attention to particularly egregious examples of what has been called “patient dumping.”^11^ ^,^ ^12^ In response to these concerns, in 2019 the California State Legislature enacted into law Senate Bill 1152 (SB 1152), a unique legislative mandate that requires a written plan to coordinate medical and social care upon discharge of PEH from hospital emergency departments (ED) and inpatient settings. Until its temporary suspension in March 2020 due to the COVID-19 pandemic, the law required hospitals to meet the following criteria to maintain licensure^13^:

1. Effective January 1, 2019, hospitals must offer and document services prior to discharging any PEH. These services include providing a meal, weather-appropriate clothing, referrals, medications, appropriate infectious disease screenings, and vaccinations; contacting the primary care clinician or coordinated entry system; conducting health insurance screening; and transporting the patient to the discharge destination within a 30-mile or 30-minute radius of the hospital.
2. Effective July 1, 2019, hospitals must create a written plan for care coordination between behavioral health, social service, healthcare, and appropriate non-profit service agencies. Hospitals must also maintain a log of discharged PEH with their discharge location and evidence of completing the discharge protocol.

To date, California is the only state to have passed such a law. However, the Healthcare Association of Hawaii implemented discharge guidelines akin to California’s in anticipation of a similar proposal passing through the Hawaii State Legislature.^14^ Understanding the implementation and impact of this law on hospital procedures (and ultimately on patient outcomes) is critical both to California’s future investments in this area and to other states considering similar legislation to improve the health of PEH. This qualitative study begins to address these evidence gaps by exploring the following research questions:

1. What are hospital staff and leaders’ perceptions of SB 1152 and the law’s impact on hospital discharge processes?
2. What are the principal facilitators and barriers hospitals have faced in meeting the law’s requirements?

## METHODS

We conducted a qualitative research study using semi-structured interviews with key informants. Key informants, defined as individuals involved in the implementation of SB 1152, included leaders, managers, and frontline healthcare workers from hospitals subject to the law. We focused on two California counties that we anticipated would be strongly impacted by the legislation: Humboldt County, which in 2019 had the highest per capita rate of homelessness in the state, and Los Angeles (LA) County, which had the highest number of individuals experiencing homelessness in the state.^15^ ^,^ ^16^ Because Humboldt is a rural, northern county and LA County is a mostly urban Southern California county, this approach also offered an opportunity to understand the law’s impacts in geographically diverse settings. In both counties, study staff used emails and phone calls to reach leaders of general acute care hospitals with EDs. In Humboldt County, we recruited at least one participant from each of its four hospitals. In LA County, 69 hospitals met our inclusion criteria, and the local hospital association also circulated our study invitation. Key informants from 10 LA County hospitals agreed to participate.

After interviewing the first key informant at each hospital, we used snowball sampling to recruit additional participants. Participant outreach efforts included a maximum of three rounds of follow-up emails or phone calls. The study protocol was approved by the Institutional Review Board at the University of California, San Francisco.

Between September 2020–May 2021, a medical student (HA) trained in qualitative research methods conducted 24 interviews with 28 participants. Interviews lasted approximately one hour, were conducted via Zoom (Zoom Video Communications, Inc, San Jose, CA) and recorded. Interviews were conducted with each participant individually when possible; four interviews were conducted in dyads to accommodate informants’ schedules. For all conducted interviews, HA developed and used an interview guide specific to this study (see Appendix A), which included questions about hospital protocols in place for PEH prior to the enactment of SB 1152; changes made after the law was enacted; perceived implementation factors, including the impacts of COVID-19; and overall impressions regarding discharge planning for PEH.

During hospital recruitment, our team became aware of a concurrent research effort focused on SB 1152 that was being conducted in LA County between June 2020–March 2021. That study was a mixed-methods evaluation that combined quantitative analysis of data extracted from the electronic health record, manual chart review, and interviews with patient-facing clinicians and staff.^17^ That concurrent study focused only on county-affiliated hospitals, three of which met our inclusion criteria. There was considerable overlap between the study goals and the interview guides used in both research projects (see Appendix B). Based on these similarities and to minimize the interview burden for participants from the LA County-affiliated hospitals, the teams developed a shared data use agreement that enabled us to review transcripts from semi-structured interviews conducted with eight leaders at three additional hospitals in LA County. Investigators from the other study joined our study team as collaborators in data interpretation and co-authored this paper.

In summary, we conducted 24 interviews and received access to eight additional interviews for a total of 32 interviews from two California counties across 16 participating hospitals (Table 1). The number of participants from each hospital ranged from one to five. All interviews were anonymized, professionally transcribed, and analyzed using qualitative analytic software Dedoose version 9.0.17 (SocioCultural Research Consultants, LLC, Manhattan Beach, CA).^18^ HA and a senior research associate on the study team, YC, developed a preliminary codebook by open coding the first four transcripts together. Subsequently, HA analyzed the remaining transcripts and reapplied new codes to previous transcripts; YC reviewed the coded excerpts. Both team members refined the codebook through weekly reconciliation and analysis meetings and received feedback from other team members. There were no significant discrepancies in the code application.

**Table 1.** Summary of participating hospitals.

	Non-profit	For profit	University owned	County owned	Total
Humboldt	2 hospitals	1 hospital	—	1 hospital	4 hospitals
Los Angeles	5 hospitals	2 hospitals	2 hospitals	3 hospitals* *data collected from LA county study	12 hospitals
Total	7 hospitals	3 hospitals	2 hospitals	4 hospitals	16 hospitals

Throughout the process, we also reflected on how our backgrounds and perspectives influenced our interpretation of the data. We used the Consolidated Framework for Implementation Research (CFIR) as a framework to build the interview guide and to guide our thematic analyses of interview data. This consisted of reviewing the applied codes to generate analytic memos for each hospital, which were then synthesized into analytic memos that reflected each construct within CFIR. The CFIR identifies constructs across five interactive domains to influence implementation effectiveness: 1) outer setting; 2) inner setting; 3) intervention characteristics; 4) individuals involved; and 5) implementation process.^19^ In this study, the *outer setting* included community resource availability, the impact of COVID-19 on such resources, and guidance by government agencies. *Inner setting* included hospital characteristics and internal resource availability. *Individuals involved* focused on staff roles with regard to SB 1152 and staff perceptions of social care for PEH. *Intervention characteristics* focused on perceived positive and negative impacts of SB 1152, and the staff’s beliefs about the law itself. Finally, the *implementation* *process* domain focused on the execution of changed workflows, initial responses to the law, and coordination among staff and hospitals.

## RESULTS

### Overall Perceived Impact of SB1152

While participants described many shortcomings to SB 1152, when asked about their overall perceptions of the law, many shared positive perceptions. They noted that being held accountable by the state law prompted a wide range of hospital changes that helped to systematize discharge planning for PEH (Table 2).

**Table 2.** Perceived positive impacts of Senate Bill 1152: examples from key informant interviews.

Perceived positive impacts of SB 1152 on hospital processes	
Increased accountability and consistency in documentation and service delivery	“I feel as if there has been a difference between before SB 1152 and now. In that there’s much more accountability in terms of all individuals that are touching a patient throughout their stay. Whether it’s the doctor, the nurse, the licensed vocational nurse or certified nurse assistant. Then social work and case management. There’s definitely more accountability.” – Clinical Social Work Supervisor, non-profit, LA County “I think our biggest learning curve was just how we were tracking and documenting the individuals that were presenting into the hospital, where before that was kind of hit or miss if we even asked them if they were homeless.” – Manager of Care Transitions, non-profit, Humboldt County “I don’t want to be super critical of SB 1152, because I think it gives guidance and I think it helps. And I like the collaborative effort that it really does pull different services responsible to make sure that they have clothing and they have some food and they have their immunizations that they need. I think these types of bills are very necessary to make sure that there’s some accountability. But at the same time, we need to work with the community as well.” – Clinical Social Work Supervisor, county, LA County “[SB 1152] probably put [discharge planning] more to the forefront and kind of forced us to evaluate every one of our discharges for homeless [patients] to make sure they’re safe. So, I can’t say that that’s a bad thing.” – Director of EM and Trauma Services, non-profit, Humboldt County “We’re proud of the fact that we’re very consistent. If we do it once, we do it 100 times and everybody gets the same” – ICU/ED Nurse Manager, for profit, Humboldt County
Improved quality of resources	“We also wanted to make sure that everyone had an identified place for their clothing… we actually ordered clothing from a local vendor who offered it at a discounted price. What ended up happening is we were able to provide that vendor’s contact information to all of the other ministries. Now that is our contact for all six to eight ministries to order their clothing. It’s weather appropriate clothing. T-shirts, sweatshirts, sweatpants, sweat shorts, socks, shoes. We also have ponchos and underwear. The essentials basically.” – Clinical Social Work Supervisor, non-profit, LA County “Another change for us is that we provide more cab rides. We always provide bus tickets, but now we will provide a cab ride just depending on their situation. If [patients] are having a difficult time with accessing the bus, then we provide cab rides, and we meet the [SB1152] criteria [of providing transportation] within a 30-mile distance.” – Social Worker and Nurse Case Manager, for profit, Humboldt County “SB 1152 made us more responsible for making sure the patient gets their medication. So when the pharmacy is open, we’ll fill them and make sure that the patient has them in hand when they leave, … even after hours now…rather than just [giving] them a prescription and say[ing], ‘Go to the next free clinic and go get it filled.’. [Instead], what patients are being told is, come back to the emergency room in the morning, the social worker will help you get it filled… So that was one thing that SB 1152 did for us, made us make sure that our patients have the proper treatment and prescriptions filled.” – ED Social Worker, university, LA County
Streamlined processes	“I think what’s changed is there’s a lot more tracking and a more streamlined approach to it, and also now it’s the hospital or the nursing staff or physician initiation [to provide services], rather than patient requesting for services.” – Nurse Manager, non-profit, Humboldt County “And the Box…setting up this resource system for everybody that’s much more friendly to navigate and we’re updating it always in real time has really helped to streamline resources and update resources.” – Associate Chief of Clinical Social Work, university, LA County
Improved awareness of homelessness	“Through this law, we realize more that there’s people that live in their cars, that are couch surfing. When they come to the hospital they might look like a normal patient, they have proper clothes…like there’s nothing wrong with them. But then when we look into their story, then we find out they’re living in their car, they’re just bouncing between friends…it brought the spotlight into this population, and even if [people like hospital security guards] don’t know the specifics of the law, people [at the hospital] know that someone’s required to do something.” – ED Social Worker and Homeless Care Coordinator, university, LA County
Respect and funding for social care staff	“It made it to where we have more support to do our job from our own organization…I think that we have more professional respect in what we do.” – Clinical Social Work Supervisor, non-profit, LA County “I think it was the pressure of SB 1152 that came that made [our hospital administrators] say, okay, we really should look at this [request for hiring homeless care coordinators], and…get on board with that.” – ED Social Worker, university, LA County

*SB 1152*: California Senate Bill 1152; *LA*: Los Angeles; *ED*: emergency department.

#### Increased accountability

Many participants noted that their hospitals had already established protocols for some of the requirements of SB 1152 prior to the enactment of the law (such as providing meals and clothing and linking patients with community resources). However, the same participants remarked that the law increased staff accountability to ensure that PEH were more consistently identified and provided with resources prior to discharge.

#### Improved quality of resources

Participants noted that prior to SB 1152, the quality of discharge resources offered to PEH did not consistently meet the law’s standard. The law’s requirements led staff to standardize both the provision and acquisition of resources. One hospital, for instance, changed the type of clothing being provided and improved the distribution efficiency by using its materials management department. Three other hospitals began an initiative to collect clothing in bulk from local vendors. In another example, to comply with the requirement to provide appropriate medications before discharge, two hospitals developed new protocols for patients to obtain medications from the hospital pharmacy, which was a change from pre-SB 1152, when they had been referring PEH to local free clinics.

#### Streamlined processes

Many participants also noted that SB 1152 led to more streamlined discharge processes. Two hospitals developed a centralized, up-to-date database of information on shelter options and community programs for social workers to use during discharge planning. In contrast, another hospital developed accessible resource packets that provided similar information for patients. Participants from two other hospitals reported that SB 1152 led them to make improvements in their referral systems (one began using a centralized calling system, and the other developed a shared online resource folder) that helped staff procure beds for PEH in shelters and recuperative care centers.

#### Improved awareness of homelessness and social care staff

Informants also expressed that the law led staff to better appreciate the complexity of issues about homelessness and the role that hospitals can play in helping address some of those issues. Furthermore, several social workers felt that the law strengthened the respect and support they received at their hospitals. In one hospital, informants indicated that the law served as a catalyst for funding new social care staff specifically to coordinate care for PEH.

In parallel, participants shared perspectives about the negative impacts of SB 1152. This included increased staff burden and consumption of hospital resources, and limitations in the scope of the law. (Table 3).

**Table 3.** Perceived shortcomings of California Senate Bill 1152: examples from key informant interviews.

Perceived shortcomings of SB 1152	
Increased initial staff burden and stress	“The biggest panic came from the social workers who work in the ED. They hit the ground running. So, it was quite overwhelming for them for a while. We had to do a lot of care in there just to calm the nerves.” – Care Coordination Director, Non-Profit, LA County “The IT people had to build into the nursing progress notes, the whole part about homelessness. It was rough in the beginning, but I think [nurses] do it okay now. A lot of grumbling about it like, ‘Don’t we do enough?’” – Social Worker & Nurse Case Manager, for profit, Humboldt County
Increased utilization of resources and time	“We do as much as we can, but some of the testing and the assessment that we do are maybe wasteful, because it’s a repeat of everything, but it’s a new presentation. So, the physician has to do everything, the testing, we do lab work, and the whole nine yards. So, I don’t know if some of that is redundant and wasteful.” – Social Work Manager, non-profit, LA County “Again, we have a lot of homeless people. So, it’s gotten to the point that our social worker and our seasoned staff kind of know all our homeless people that visit the ED frequently. And they kind of know that they’ll either accept or deny whatever resources we have to offer. So, it’s almost to the point where we already know what they’re going to say as soon as we see them. And, you know, I mean, but we still have to go through hoops. It is a mandated requirement.” – Nurse, county, LA County “We have a lot of homeless populations showing up in our emergency rooms. Some of them are pretty savvy with the Senate bill, so we have to provide food and clothing and then find a destination point. And so our resources get heavily consumed, going through this populous of patients… But they have [been savvy] even without the Senate bill.” – CNO, non-profit, Humboldt County
Limited scope of the law	“Say, there’s a homeless person, police will pick them up, bring him to the hospital, drop them off. And it’s really not an appropriate place to drop off… in [theory], [SB 1152] is a good idea, but it really [is] a Band-Aid. Because it sticks the hospital with making this plan, and the hospital is really not an appropriate place to make a long-term plan for someone. Even when we connect the patient to…agencies…it’s still lacking. Mental health is a huge piece that’s missing because we don’t have the resources to really treat people like we should treat them. So, I think really, what we’re just creating is like the cyclical, patient relationship with the hospital. We do our discharge properly, they possibly get housing if they wanted, many say no… a lot of times, they don’t fit the criteria to go to these recuperative cares, and then they end up coming back again.” – Clinical Social Worker, non-profit, LA County “[The law] helped with something more immediate. But it didn’t really help with something long term, which I think is a drawback with the law…there could be other long-term solutions that may need to be addressed.” – Social Worker, county, LA County “SB 1152, I think comes from the feeling that we need to intervene and we need to hold somebody accountable. And the hospitals in this case are the ones that were chosen to be accountable. Do I really think that’s the answer? No, but do I think that we can and should be involved? Yes. So that’s where it stands.” – Nurse Administrator, county, LA County “[I want the state legislators] to know that it’s an interdisciplinary approach, and then it’s not just something that we could fix in the hospital. We have to be able to work with community partners and just along the continuum of care to meet the need. So, I think that if hospitals are held to such a high standard, then I feel like every other agency before and after should be held to a high standard.” – Social Work Manager, non-profit, LA County

#### Increased initial staff burden and stress

Participants across hospital departments and positions described an initial increase in stress, reluctance to participate, and concerns about the division of responsibilities required under SB 1152. They reported lack of clarity about which staff members (eg, social workers, nurses, or other staff) would be assigned the different requirements outlined in the law. Some reported hesitancy about assuming new tasks since they already felt overburdened with existing responsibilities. Still, those we spoke with reported that most of these concerns abated after the initial phase of their hospital’s implementation efforts.

#### Increased hospital resource consumption

Participants also expressed concern that the law’s requirements would lead to hospital resource strains. Interpretations of SB 1152’s requirements led some hospitals to conduct the full discharge protocol for each PEH encounter regardless of how recently the patient had last presented, which some informants, especially in the ED, noted was time consuming and redundant. Others anecdotally noted that SB 1152 led to an influx of patients using the hospital for social services and were concerned about the increased consumption of hospital resources and staff time. While the resource and time constraints were a concern across most hospital informants, several participants suggested alternative explanations for the law’s impact on patient numbers. They suggested that the perceived influx of patients may have reflected an increase in homelessness in the county overall, increasing medical complexity of PEH, and lack of access to social services in the community, rather than being a consequence of SB1152. As one social worker noted:

*“[SB1152] has made the ED very impacted because homeless people will come and say, ‘I know I can get resources here.’ Which I get it…we’re open 24 hours a day, and it’s a one-stop shop for everything that you need, and no one’s going to turn you away, versus having to go to one of the community centers, and stand in line or possibly be turned away… It also goes back to if more time and resources can be put into making the community resource centers better, it would create a better flow and a better system for us.” (ED social worker, university, LA County)*

#### Limited scope of the law

Participants expressed concerns that SB 1152’s narrow focus on hospital discharge processes overlooked broader, community-level barriers to addressing homelessness, such as the lack of affordable housing and poorly coordinated systems of care for PEH. They described how this contributed to difficulties in implementing changes to meet the law’s requirements and to frustration among staff that their work amounted to providing only short-term solutions to meet the very complex needs of PEH.

### Implementation Barriers and Facilitators

Various factors influenced hospitals’ capacity to make changes to meet the law’s requirements (Table 4). Participants noted that discharge processes for PEH were facilitated by strong community partnerships with shelters and sober living houses, as well as by Medicaid plan coverage for recuperative care costs. However, a more consistent barrier that emerged was an overall lack of community resource capacity, particularly in mental health facilities, discharge locations (skilled nursing facilities [SNF], recuperative care centers, shelters), long-term housing, and navigation centers. In rural Humboldt County, community facilities were scarce; in LA County, these facilities existed but were already over capacity and functionally inaccessible. In both counties, lack of capacity limited hospitals’ ability to provide discharge location options for PEH. Additionally, interviewees from both counties described the specific challenge of getting PEH accepted into SNFs, as *“SNFs have the ability to say no,* *just because they don’t want to deal with the process of trying to safely discharge them.” (Clinical social work supervisor, non-profit, LA County).*

**Table 4.** Implementation barriers and facilitators: examples from key informant interviews.

Implementation barriers	
Limited community resources	“We still are dealing with lack of resources in order to satisfy the law. I think even after SB 1152 was [put in place], it was as if nothing had changed. We still have the same limited women’s shelters, men’s shelters. [We need] more recuperative care [and] more long-term housing options… those options should have been available as of January 1st, 2019. It’s almost like having family come over for Thanksgiving but all you have is Top Ramen and half a jug of water in your refrigerator. Like, okay. Well, do what you can, you know.” – Clinical Social Worker, non-profit, LA County “Eureka has [a] psychiatric hospital…and I think it’s only 16 beds. However, it’s the only one from Santa Rosa to Brookings, Oregon. That’s four hours in each direction. There are no towns around us to absorb it. It’s all wilderness between north, south, east, then there’s the ocean.” – Social Worker and Nurse Case Manager, for profit, Humboldt County “For someone who is experiencing homelessness, those patients are just much, much harder to find a place for, because facilities don’t want to accept them, unless they know in the beginning that there’s a discharge plan waiting for them at the end of their course there.” – ED Social Worker, non-profit, LA County
Limited hospital funding	“So, it’s like one size does not fit all and small rural facilities, especially like ours, we’re a privately owned for-profit. We ride the ragged edge of financial disaster every single day and sometimes we can’t afford to buy [even] angiocaths. So, kick a little money our way… And the cudgel that you want to beat these large urban centers with, is just like Godzilla’s footprint on the small rural facilities.” – ICU/ED Nurse Manager, for profit, Humboldt County [SB 1152] has caused hospital more money in some way because we do the increase in number of meals, each meal may cost $10–$12 because it has to be a meal, not a sandwich. So, you know, and then when you multiply by 10 to 20 and 365 days, that could add up.” – ED Physician Administrator, county, LA County
Limited staffing	“But I would say one of the main limitations is just the fact that we don’t have 24-hour social work and case management… and there are only two acute medical social workers. They can’t always call when we have a homeless patient who’s discharging.” – Clinical Social Work Supervisor, non-profit, LA County
Limited state support	“[The] law is up to interpretation…it will be nice to clarify if this was intended for inpatient [discharges]. And then what are some of the things that need to be done from the emergency department. What about urgent care, what about from the clinics[?] Clinics…they don’t follow any of these [SB 1152] rules, or even urgent care, while the patient goes to [the] ED then [for us] to do things, including making arrangements for transportation and document all this need. So, I think the clarification of the law would help.” – Emergency Physician Administrator, county, LA County “I guess for me personally, a better understanding of, if someone doesn’t want medication, are we still obligated to get it to them? There are some questions we still have…where I get tripped up a little bit is like, well, how much are we supposed to bend over to get someone medication if they don’t want it? Can we just say, we don’t need to do that if they don’t want it? That’s the one hiccup that I get chipped up about.” – Clinical Educator, university, LA County “Well, maybe a toolkit of ‘Oh, these are options’ could have been [helpful]… It’s like, ‘Here’s what hospital A is doing and has done, and this meets our criteria. Are you doing this? Here’s some ideas.’ Something like that probably would have been helpful.” – Manager of Care Transitions, non-profit, Humboldt County
Implementation facilitators	
Strong community partnerships	“We continue to have strong partnerships with the local rescue mission and a number of sober living houses and places like that, where patients could go at discharge.” – Nurse Manager, non-profit, Humboldt County “We’ve also established some really great relationships with community entities that really worked to address homelessness in the South Bay. We have one entity called Harbor Interfaith Services that recently opened up two Bridge Home sites, which is interim housing. They’ve been really diligent in making sure to keep in contact with us to identify some of our homeless individuals that are constantly coming back to the ED and the hospital. That way they can get them into the Coordinated Entry System and get them connected to long-term housing…They’ll come to the hospital. Assess the patient. Put them in the Coordinated Entry System…Then we can potentially have them discharged to that interim housing. As opposed to discharging to an emergency shelter or back to the streets.” – Clinical Social Work Supervisor, non-profit, LA County
Donor funding	“There should be more support…we’re lucky that we’re at [this hospital], and [we have] funding for us to pay for recuperative care. We’ve been using recuperative care to place the homeless, but the hospital’s paying for that. For the hospitals that don’t have that much money, they don’t have that luxury.” – Homeless Care Coordinator, university, LA County
Hospital staff for PEH	“Everybody should have a point person that’s building the relationship and really has the bandwidth to get out there for the resources. Adding [the homeless care coordinator] has been the best thing that’s happened for us. Just all around, because he’s been able to make the relationship within the community, and really tell us, ‘No, this is an existing resource, this doesn’t work,’ so we’re all in touch and not out of date.” – ED Social Worker, university, LA County

*SB 1152*, California Senate Bill 1152; *LA*, Los Angeles; *ED*, emergency department; *CNO*, chief nursing officer; *ICU*, intensive care unit.

The coronavirus 2019 pandemic exacerbated community resource constraints in both counties, posing another barrier to safely discharging PEH. While many participants noted the initial increase in resources as a result of initiatives such as Project Roomkey,^20^ one participant noted that by pandemic year two, those resources were no longer available.

Another significant implementation barrier was the limited funding for required services and social care staff. Without funding, hospitals had difficulty covering expenses, including for patient clothing, food, and transportation vouchers. Many hospitals also noted difficulty covering recuperative care costs, which hospitals were forced to absorb if the patient was unable, or insurance declined, to cover related expenses. While many hospital leaders in both counties expressed concern about hospital resource limitations, informants from smaller hospitals in Humboldt County more strongly emphasized the negative impacts of the service and staffing shortages.

Nurses and social workers from one of the Humboldt County hospitals reported sometimes paying out of their own wallets to provide supplies for PEH at discharge, including for items such as tents, sleeping bags, blankets, and backpacks. Not surprisingly, hospitals that endorsed institutional resources to support social care commitments for PEH reported that this support facilitated efforts to meet SB 1152’s stipulations. Examples included having a homelessness task force and staff explicitly hired (eg, homeless care coordinators) to ensure patients were discharged with appropriate resources and referrals to community agencies. Yet even in these instances, many interviewees underscored that they could not hire or maintain adequate staff to continue meeting the law’s requirements. The lack of staff capacity delayed discharges for PEH, particularly when discharges were outside regular business hours.

Furthermore, although the California Department of Public Health and professional organizations such as the California Hospital Association offered some guidance to hospitals about the law’s requirements,^21^ other barriers noted by participants in both counties related to ambiguity about the law’s requirements. The ambiguity contributed to different interpretations of the law. For example, one hospital noted that they decided that the discharge requirements would not apply if a patient had been discharged from the same hospital within the prior 48 hours. Another hospital chose to follow the discharge protocol each time a PEH was hospitalized or in the ED.

The law’s ambiguity also confused community partners and patients. According to participants from five hospitals, some community advocates and patients initially misinterpreted the statute as requiring hospitals to provide housing when needed, leading at least one hospital ED to experience a surge of housing requests from PEH. In that more rural hospital, a participant described, *“There was definitely a learning curve, and dialogue had to happen with community members too.” (Manager of Care Transitions, non-profit, Humboldt County).* While this was less of a concern for participants compared to the lack of community resources and funding, the law’s ambiguity still added complexity to the implementation process for many hospitals.

## DISCUSSION

SB 1152 is a novel California law that aims to improve health outcomes for PEH by mandating standardized hospital discharge protocols. Mandates for care delivery specific to PEH are unique; prior government-led efforts in this realm have focused on other ways to support PEH using Medicaid expansion and programs such as Healthcare for the Homeless.^22^ ^–^ ^24^ Our findings highlight that SB 1152 had several positive effects, including more systematic discharge processes for PEH, increased awareness of and accountability for addressing homelessness, and increased support for social work in some California hospitals. However, our study informants also shared critical concerns that affect implementation and sustainability, including concerns about the lack of funding for hospital social care staff and related services, insufficient state guidance about the law’s provisions and enforcement, and limited investments in community resources that are needed to support PEH.

Although little data specific to SB 1152’s impacts have been published, our nuanced findings are consistent with overarching findings from the mixed-methods study in LA County.^17^ That study revealed several barriers to SB 1152 implementation, including resource limitations in hospital and community environments and ongoing ambiguity about the bill’s requirements. Our study included different types of hospitals and more informants across two counties with different resource capacities, yet it underscored the same significant implementation barriers. Future policymaking can address these concerns by 1) increasing hospital funding for social care services, 2) strengthening implementation guidance, and 3) better integrating healthcare mandates with efforts to expand available community-level resources for PEH.

### Increase Hospital Funding

Successful implementation of state-level initiatives requires financial resources. For example, California’s WPC pilot program was funded under a Medicaid 1115 waiver, and implementation studies of the program have concluded that its success was contingent on adequate funding and community partnerships.^25^ ^,^ ^26^ In contrast to funded programming, unfunded legislative mandates often lead to increased financial strain on health systems. A salient example is the Emergency Medical Treatment and Active Labor Act (EMTALA), which requires hospitals to provide emergency care to patients regardless of their ability to pay.^27^ While EMTALA has led to improved emergency care for vulnerable populations, compliance across hospitals has varied in part due to the financial challenges of providing uncompensated care.^28^ ^–^ ^30^ Similarly, our study of SB 1152 highlights challenges hospitals face implementing new protocols without new funding. Attaching funding to SB 1152’s requirements would enable hospitals to cover costs associated with hiring more social care staff and obtaining needed resources for PEH, including meals, clothing, transportation, and recuperative care beds.

### Strengthen Guidance, Education, and Training

In addition to highlighting funding needs, many informants emphasized that implementation would have been streamlined with more guidance, education, and training about the law and strategies for meeting the mandate’s requirements. This echoes findings from a systematic review on common hospital implementation barriers^31^ and hospital experiences with other mandates: for instance, complaints about EMTALA’s ambiguity similarly posed barriers to initial implementation efforts.^30^ ^,^ ^32^ In the case of SB 1152, informants suggested that the state offer more guidance on how frequently to conduct screening for homelessness, what screening measures should be used, and the appropriate intensity of interventions. As data accrues, these supports should include detailed information about best practices (eg, toolkits), which can help standardize hospital practices and allay hospital concerns about compliance. Training and education materials about the law should also be directed to community advocates and resource centers to ensure communities are accurately informed about the law’s requirements.

### Facilitate Action on Upstream Solutions

Finally, while laws like SB 1152 ideally will improve hospital discharge processes, healthcare experiences, and outcomes for PEH, study participants emphasized that hospital-focused policies enacted without simultaneous expansion of community resources are inadequate for meeting long-term, complex needs of PEH. These findings are consistent with scoping reviews that describes how integrated community care and support services are critical to improve outcomes for PEH.^33^ ^,^ ^34^

To move in this direction, any legislation intending to improve care for PEH must be accompanied by the expansion of community-based health and social service resources across the state—both in rural areas where these resources are scarce and in urban areas that may have resources that are over capacity. Discharge facilities such as SNFs, recuperative care centers, and shelters are sorely needed. These institutions must also be held accountable for accepting PEH who require care; that accountability is likely to require new policies, such as Medicaid reimbursement reforms or coverage mandates. Expansion of psychiatric facilities, sobering centers, and general navigation centers can also help to reduce the reliance of PEH on ED services and, concurrently, improve care and outcomes post-discharge for PEH. Overall, reforms and policy incentives across other sectors that have many touchpoints with PEH are necessary to better support well-meaning initiatives like SB1152 and address the long-term, complex needs of PEH.

## LIMITATIONS

Findings should be interpreted considering three key study limitations. First, there may be selection bias as we interviewed informants who responded to our outreach attempts and thus may have been more likely than non-respondents to hold strong opinions about SB 1152. However, to mitigate selection bias, we conducted multiple outreach efforts and relied on hospital associations to circulate our study invitation to hospitals that met our inclusion criteria. Second, our findings may be influenced by the fact that some study data came from a concurrent study in LA County. However, prior to incorporating the LA County data, the analysis team reviewed all transcripts to ensure that the same topics had been covered at a similar level of detail as done in the primary study. Other published research has also combined data from similar studies when the content was similar.^35^ As a result, we believe the addition of the concurrent study data enriches this study by increasing the number of knowledgeable participants. A third potential limitation is that enforcement of SB 1152 was suspended in March 2020 due to the COVID-19 pandemic. Therefore, we included questions in the interview guide that focused on pandemic-related protocol changes; most informants indicated that the pandemic did not lead them to abandon their SB 1152 protocols.

## CONCLUSION

This study provides insight into the implementation process and perceived impacts of SB 1152 from hospitals across Humboldt County in northern California and Los Angeles County in the south. Future research should aim to examine the law’s impacts on a broader array of hospitals and how PEH have personally experienced hospital changes. Overall, SB 1152 helped hospitals focus on the safe discharge of PEH. But high-quality care for PEH will also require more community resources and other care system investments. While hospitals found creative ways to interpret and implement this unfunded mandate, they faced significant challenges in meeting the law’s requirements. Future policies that refine or expand on SB 1152 to improve care for PEH should focus on strengthening implementation supports, including funding, training, community investments, and reforms both within and outside of health systems.

## Supplementary Information




