# Supplementary material for: Implementation and Impacts of California Senate Bill 1152 on Homeless Discharge Protocols
Source: West J Emerg Med. 2023 Nov 8;24(6):1104–16. doi: 10.5811/westjem.60853 (PMC10754197; doi:10.5811/westjem.60853)
Supplement: Supplementary file 1 [file wjem-24-1104-s001.docx]

**Appendix A.** Interview guide for primary study.

| 1. General *(5 minutes)*   - Can you briefly tell me about your role (title, responsibilities)? - How long have you been at this job? - What is your role related to homeless discharge?   Moving on to talking about <hospital name>,   - How is the hospital system structured? - Is there anything important for us to know about the hospital system? - Could you tell me a little bit about the type of patients you serve across the different hospitals in your system? - How many of your patients are homeless? - Do you think your hospital system sees more homeless patients as compared to other hospitals in the county? |
| --- |
| 2. SB 1152 overview, related services + changes made with SB 1152 *(20 minutes)*  So now we’ll shift gears and talk more about Senate Bill 1152. Are you familiar with that law?   - For starters, when and how did you first hear about this law? - What was your initial reaction / admin’s initial reaction to the law? Do you think differently now? - Prior to SB 1152 becoming a law, what kinds of services, policies, or plans did your hospital/system already have in place for patients experiencing homelessness? - So then, SB 1152 passed in November 2018. What changed when the law went into effect? Walk me through - what did you do differently at the systems level?   *Probes*   - *Were these protocols on paper or were they actually implemented?* - *How were these services coordinated?*   - *When would they occur?*   - *Who was responsible for these services?* - *How were they funded? Where does that money come from?* - *How/why did you implement these services to begin with?* - *What were the outcomes and limitations of these services?* - *So you mentioned how SB 1152 changed your workflow. Why did SB 1152 make things change? What about SB 1152 helped facilitate that/made you do that? (Ask after each change, if relevant.)* |
| **3.** Facilitators and barriers to SB 1152 *(10 minutes)*  *Initial response*   - Walk me through the steps of getting this program started.   *Probes*   - *What were the first steps?* - *Who were the project leaders?* - *Were new staff hired?* - *What technical assistance was needed?* - *Was any new technology developed?* - *Did you develop any new partnerships with community-based organizations?*   *Workflow changes, cost*   - How did the changes you mentioned affect the workflow of the hospital system?   - What was working about these new initiatives and what was not? - Did implementation of these changes affect hospital system costs? If so, how? If not, why not? - *(IF system leadership)*: How did your different hospitals react differently to SB 1152? Was it easier or harder for some to implement changes to comply with the law? What in your opinion made it easier or harder?   *Benefits and challenges*   - What is your general take on SB 1152? Good idea/bad idea?   - Was it helpful in improving or coordinating care for homeless patients? How?   - What worked with this policy?   - What would’ve worked better?   - Do you have any experience with other policies that worked better? |
| 4. COVID-19 and SB 1152 *(10 minutes)*   - How, if at all, did the COVID-19 pandemic affect the decisions you had made about the requirements of SB 1152? - What were some new decisions / changes you had to make about homeless patient discharge processes because of COVID-19? - Did any parts of what you had put into place to comply with SB 1152 help you in your response to COVID-19? - Do you think there’s anything about how you responded to the pandemic that will affect your hospital’s preparedness to implement the requirements of SB 1152 in the future? If so, how? If not, why not? |
| 5. Overall impression/conclusion *(5 minutes)*   - In general, how would you characterize the attitude or culture among other staff in this hospital regarding PEH?   - How do you think COVID-19 changed this, if at all?   - Why do you think that might be the case? - The law was enacted in 2019, and it’s been about two years. Looking back at everything that’s gone on with SB 1152, what do you think can be improved in homeless discharge planning?   - How can the state support you/your hospital in this process?   - Are laws like SB 1152 the best way to support you/your hospital? - We’ve heard that some other states are looking at what California did with SB 1152 and thinking of passing similar laws. What would you want them to know, based on your experience with SB 1152? - Before we finish, is there anything we haven’t talked about yet that you feel is important for us to understand how SB 1152 is being implemented in your hospital? |
