# Supplementary material for: Implementation and Impacts of California Senate Bill 1152 on Homeless Discharge Protocols
Source: West J Emerg Med. 2023 Nov 8;24(6):1104–16. doi: 10.5811/westjem.60853 (PMC10754197; doi:10.5811/westjem.60853)
Supplement: Supplementary file 2 [file wjem-24-1104-s002.docx]

**Appendix B.** Interview guide for concurrent Los Angeles County study.

| 1. General   - Title in the clinic/department   - Clinician (MD/NP)   - Director   - RN/NA   - Social work → clinical social work supervisor   - Other If other, list_________________ - Inpatient/outpatient/ed/urgent care   - If outpatient, primary care or specialty care {Full time administrative or pt care also?} - How many years have you worked in health care? _______ - How many years have you worked in your current position? |
| --- |
| 2. SB 1152 overview, related services and changes made  *For frontline workers: ask how screenings were introduced to them (training, informal, changes to workflows, how they feel about this new task, how this could have been done better) instead of asking about how screenings were planned.*   - Could you please walk me through how the process in your clinic works currently? (When are the patients screened, who is screened, by whom, who reviews the results, how are they connected, where is it documented, who manages the data, is there any follow up for referrals, etc?) - Please tell me about the decision-making process for how your team/ health center decided to begin screening for social needs. What team members were involved in the development? - How did you choose which social determinant(s)/social need(s) to screen for and address?   How did you choose your screening tool?   - Please tell me how you identified social determinant(s)/social need(s) referral sources? Did you go through a vetting process to decide which agencies to refer to? - (if this hasn’t been discussed in questions 1-3) Did you talk to patients prior to implementation about screening and referral to determine their willingness to be screened/referred? Have you gotten feedback from patients since (formal or informal) on the screening and referral process? |
| 3. Facilitators and barriers to SB 1152   - When you first implemented the project, what barriers did you face for implementing the screening? (Examples staffing, staff buy-in, changes to workflow etc) How did you overcome these barriers? Please describe any barriers that were not “overcome”? Were there facilitators that helped you establish the process? - When you first implemented the project what barriers did you face in terms of resources to connect patients when a need was identified? Please describe any barriers that were not “overcome”? What facilitators made the process easier? - Do you have funding for the project? Can you please describe what kind of staffing you have dedicated to the project and their percent effort? (for both the screening and the connection portions) - What has the impact of this project been on your clinic or department? On your patients? How did you determine this “impact”? (this question is about whether there are any evaluation processes in place) Is there any tracking going on of the processes? Has this project impacted the clinic’s other workflows? - What are the biggest challenges you face in sustaining the project? (if prompts needed, perhaps mention “human and financial resources” and time) Are there any plans to expand or change the project in the future? |
| 4. COVID-19 and SB 1152   - How has COVID-19 impacted the project you described? - What have been some of the challenges to maintaining the project during COVID? How have you dealt with them? - Have you seen any differences in the patient response to these programs since COVID began? - How might the continued presence of COVID impact the ability of your clinic to sustain the project? |
| 5. Overall impression /conclusion   - What do you know now that you wish you had known when you first began this project? If someone were trying to implement this in another clinic, what advice would you give them? - Are there others with similar projects in your institution that we should speak with? Are there others within your project that we should interview? |

*MD*, medical doctor; *NP*, nurse practitioner; *RN*, registered nurse; *NA*, nursing associate.
